# Indoor fire and smoke detection based on optimized YOLOv5

**DOI:** 10.1371/journal.pone.0322052

**Published:** 2025-04-29

**Authors:** Md. Shafak Shahriar Sozol, M. Rubaiyat Hossain Mondal, Achmad Husni Thamrin

**Affiliations:** 1 Institute of Information and Communication Technology, Bangladesh University of Engineering and Technology, Dhaka, Bangladesh; 2 Keio University, Japan.; G H Raisoni College of Engineering and Management, Pune, INDIA

## Abstract

Ensuring safety and safeguarding indoor properties require reliable fire detection methods. Traditional detection techniques that use smoke, heat, or fire sensors often fail due to false positives and slow response time. Existing deep learning-based object detectors fall short of improved accuracy in indoor settings and real-time tracking, considering the dynamic nature of fire and smoke. This study aimed to address these challenges in fire and smoke detection in indoor settings. It presents a hyperparameter-optimized YOLOv5 (HPO-YOLOv5) model optimized by a genetic algorithm. To cover all prospective scenarios, we created a novel dataset comprising indoor fire and smoke images. There are 5,000 images in the dataset, split into training, validation, and testing samples at a ratio of 80:10:10. It also used the Grad-CAM technique to provide visual explanations for model predictions, ensuring interpretability and transparency. This research combined YOLOv5 with DeepSORT (which uses deep learning features to improve the tracking of objects over time) to provide real-time monitoring of fire progression. Thus, it allows for the notification of actual fire hazards. With a mean average precision (mAP@0.5) of 92.1%, the HPO-YOLOv5 model outperformed state-of-the-art models, including Faster R-CNN, YOLOv5, YOLOv7 and YOLOv8. The proposed model achieved a 2.4% improvement in mAP@0.5 over the original YOLOv5 baseline model. The research has laid the foundation for future developments in fire hazard detection technology, a system that is dependable and effective in indoor scenarios.

## 1 Introduction

Fire hazards in indoor environments are a serious safety concern because they can cause severe loss of life and property [1–3]. It is important to reduce these risks through early detection and rapid action. Traditional fire detection methods primarily depend on various sensors that detect smoke, heat, or fire [4, 5]. Smoke detectors, often using photoelectric or ionization technologies, are widely deployed in residential and commercial buildings [6]. Heat detectors, which sense a rise in temperature, and fire detectors, which detect the light emitted by fires, are also common [7]. Although these methods can be useful in certain scenarios, they often result in high false positive rates and slower response times, especially in larger or more complex environments [8–10]. Additionally, traditional sensors are limited by their reliance on physical proximity to the fire source, making them less effective in large or obstructed spaces. They also struggle to distinguish between actual fire incidents and false alarms triggered by non-fire-related phenomena, such as steam or dust [9,10]. This highlights the pressing need for more refined and efficient fire detection systems. To address this, researchers have started to investigate image-based fire detection approaches, applying advances in computer vision (CV) and cutting-edge image processing techniques. Early approaches used simple color-based algorithms to identify fire by detecting regions with characteristic fire colors such as red, yellow, and orange. However, these methods often struggled with false positives caused by objects of similar colors [11–13]. The introduction of more sophisticated image analysis techniques, including motion detection and texture analysis, improved the reliability of fire detection. These methods analyze the dynamic and structural characteristics of fire, such as the flickering motion of fire and the smooth texture of smoke [14–16]. Despite these improvements, image-based detection approach still faces challenges under varying lighting conditions and complex backgrounds [17]. The potential of CV and AI lies in their ability to address complex, real-world challenges through automation and intelligent decision-making across various fields, including fire safety monitoring [9], construction [18], and agriculture [19, 20]. AI-based deep learning (DL) models for object detection provide a promising approach to tackle these issues in detection systems [21, 22, 23]. There is a lack of indoor fire and smoke detection-based DL models [9,15,21]. Moreover, most available image datasets are outdoor-based or mixed, presenting challenges for indoor-specific model development. These existing datasets emphasized outdoor conditions, which differ significantly from indoor environments in terms of lighting, background complexity, and other variables [21,24–26]. Consequently, they may not provide the well-annotated data required for reliable indoor detection. To address this gap, a novel dataset has been created specifically for indoor fire and smoke detection.

This study makes significant contributions by introducing a novel dataset specifically for indoor fire and smoke detection. An optimized YOLOv5 (HPO-YOLOv5) model was developed through a genetic algorithm to improve detection performance. It ensures higher accuracy and less inference time in indoor environments. The Grad-CAM method is applied to produce visual explanations for the predictions via model, hence enhancing transparency and interpretability. The HPO-YOLOv5 model is evaluated towards other state-of-the-art models, exhibiting better performance. By integrating YOLOv5 with the real-time tracking algorithm DeepSORT enables real-time surveillance of fire progression, providing actual fire hazard alerts.

The key findings of this study are as follows:

- Introduction of a new indoor fire and smoke dataset for accurate detection of fire and smoke in household settings.
- Development of an enhanced YOLOv5 model with optimized hyperparameters using a genetic algorithm, boosting fire and smoke detection performance. Comparative analysis shows our model outperforms Faster R-CNN, YOLOv5s, YOLOv7, and YOLOv8n in indoor fire and smoke detection.
- Application of Grad-CAM technique as explainable AI to generate visual explanations for the model’s predictions, ensuring transparency and interpretability.
- Integration of YOLOv5 and DeepSORT enables real-time surveillance of fire development and effective fire hazard alerts.

This paper is structured as follows: Section 2 explores related work on fire and smoke-based object detection. Section 3 outlines the materials and methods, incorporating dataset preparation, and hyperparameter optimization on the model. Section 4 presents the experimental results, along with a detailed discussion of the findings. Lastly, Section 5 concludes the study, summarizing the key findings and future research directions.

## 2 Related works

Convolutional neural networks (CNNs) have become central to modern object detection, capable of learning complex patterns and features from large datasets [27]. Among the various CNN architectures, including YOLO (You Only Look Once), Faster R-CNN [28], SSD (Single Shot Detector) [29], and EfficientNet [30], YOLO emerged as a highly effective model for real-time fire and smoke detection. YOLO models, including YOLOv3 [31], YOLOv4 [32], YOLOv5 [33], YOLOv6 [34], YOLOv7 [35], and the latest YOLOv8 [36], have been successfully applied to fire detection, showing significant improvements in speed and accuracy over traditional methods. Some of these works can be grouped by the aspect of “explainable AI”, real-time tracking, methods based on YOLO algorithm or based on region-based methods, the use of optimization techniques, etc.

In order to improve fire detection tasks, Chaoxia et al. [37] proposed a Faster R-CNN color-guided anchoring technique to enhance fire detection by using fire color features for anchor placement. The accuracy of fire detection was significantly increased when Wan et al. [38] proposed SSD, which combines feature maps from multiple convolution layers to retain detailed low-level features and abstract high-level semantic information. Dewangan and Gupta [39] developed a soft-voting based deep ensemble model for indoor fire and smoke detection, utilizing four transfer learning models: MobileNetV2, ResNet50V2, EfficientNetB0, and DenseNet121.

Beyond all other object detection models, the YOLO-based model performed significantly better than other SOTA models in many cases [40–45]. Qin et al. [40] applied YOLOv3’s multi-scale detection capability, achieving through up-sampling and splicing in certain layers of network prediction. Hu et al. [41] introduced the DS-YOLO model, enhancing YOLOv7 with the DP-ELAN module and SlimNeck to expand the receptive field, improve feature representation, and balance computational efficiency with accuracy for smoke and fire detection. Kong et al. [42] enhanced the fire and smoke real-time detection algorithm for coal mines using an improved YOLOv8s model, focusing on reducing computational complexity and integrating attention mechanisms to achieve a mAP of 91.0%. YOLOv5 [43], a popular successor in the YOLO series, improved on its predecessors with architectural innovations and optimized training, still making it more suitable for fire detection than the newer YOLOv8 in some cases [44]. Yang et al. [46] developed a lightweight YOLOv5s-based network with C3Ghost, Ghost modules, and a CA module for improved fire and smoke detection, enhancing accuracy in complex environments.

Even with the progressions in YOLO models, challenges remained in optimizing their performance for specific tasks such as indoor fire and smoke detection. Hyperparameter optimization is key to enhancing DL model accuracy and robustness. Parameters like learning rate, batch size, and momentum must be carefully tuned for optimal results [47]. Falkner et al. [48] emphasized the necessity of hyperparameter optimization in DL models by showing the strengths of Bayesian Optimization and Hyperband tuner. Manual tuning is often time-consuming and may not yield the best results. Therefore, automated hyperparameter optimization techniques have gained popularity [49]. Wu et al. [50] highlighted the importance of automated hyperparameter optimization like Bayesian optimization, genetic algorithms, and random search for DL-based side-channel analysis, given the complexity of neural networks’ large hyperparameter space. One of the key techniques, Genetic algorithms (GA), are inspired by the process of natural selection and are used to optimize complex problems by iteratively improving a population of candidate solutions [51]. In the context of DL, GA can effectively explore the hyperparameter space, identifying optimal configurations that enhance model performance. Studies [52–55] showed that GA-optimized models outperform those tuned through manual or grid search methods. GA has been applied to optimize hyperparameters in various object detection models, including YOLO. Suhail et al. [56] highlighted their key contribution in using the Evolutionary Genetic Algorithm (EGA) for hyperparameter optimization in YOLOv5, improving the detection of six categories of urine sediment particles from microscopic images. So, GA-based hyperparameter optimization may be highly effective for complex tasks like fire and smoke detection to improve model robustness and reliability. However, current optimized GA models for indoor fire and smoke detection still fall short in recent scenarios.

Moreover, the black-box nature of DL models poses challenges for their adoption in safety-critical applications. Explainable AI (XAI) addresses this issue by providing insights into the model’s decision-making process, ensuring transparency and trust [57]. Selvaraju et al. [58] introduced the Gradient-weighted Class Activation Mapping (Grad-CAM) method, which offers visual explanations for the decisions rendered by CNNs. This technique helps in understanding which regions of an image contribute most to the prediction results, thereby enhancing the transparency and trustworthiness of the model. This is particularly useful in fire and smoke detection, where understanding the model’s focus can validate its predictions and ensure it responds to actual fire characteristics. However, there is a lack of transparency-checking methods in safety-critical DL models like fire and smoke detection [59].

While recent and previous DL-based object detectors have been successful in detecting fire and smoke instances in images and videos, there is a noticeable gap in research and literature regarding practical approaches for monitoring fire progression in a video to detect actual fire hazard scenarios [60–65]. A review by Jin et al. [60] and Chitram et al. [61] emphasized improvements in image and/or video-based fire detection methods using DL, highlighting the need for improved strategies to monitor and predict fire behavior in real-time. In many cases, the integration of YOLOv5 with real-time tracking algorithms such as DeepSORT [62] has proven highly effective for continuous events monitoring. Zhang Y et al. [63] used YOLOv5 for real-time detection of surgical instruments and then employed DeepSORT to precisely track the movements of those instruments across multiple video frames. Researchers also applied this combined approach to athlete tracking [64], as well as vehicle analysis on expressways [65]. Still, there is no effective approach to tracking fire progression over time in videos to detect and manage fire hazards better.

Despite improvements in DL for fire and smoke detection, several challenges exist. Achieving high detection accuracy and reducing false positives are critical issues, especially in complex indoor environments with varying lighting conditions, occlusions, background noise, and the need to detect actual fire hazards. The availability of diverse and well-annotated indoor datasets is essential for developing effective DL models that can perform reliably across different scenarios. Furthermore, an optimized deep-learning model specifically for indoor fire and smoke detection still needs to be readily available. Additionally, ensuring model transparency and interpretability using techniques like Grad-CAM remains a challenge, as it requires making the decision-making process of the model understandable and accessible to users.

## 3 Materials and methods

### 3.1 Dataset creation method

The Indoor Fire and Smoke (Indoor FS) Dataset was designed to aid the development and evaluation of fire and smoke detection systems. To ensure its usefulness in various real-world scenarios, we compiled a diverse collection of images. The images were sourced locally using a Canon PowerShot G16 and an iPhone XS camera, as well as from online platforms (YouTube, Google Images), including CCTV footage. To develop a more well-rounded dataset, real scenes were combined with synthetic images. Images were maintained in a 60:40 ratio, with 60% captured locally and 40% sourced online and synthesized. This approach showed a diverse range of image quality and perspectives, which is vital for training effective detection models. Annotation and preprocessing of the dataset were conducted using an online tool named Roboflow. Annotations were done carefully, ensuring that bounding boxes were closely enclosed around each object within the images. Each image file was paired with a corresponding.txt file containing annotated information, i.e., object class, coordinates, height, and width.

**Fig 1:**
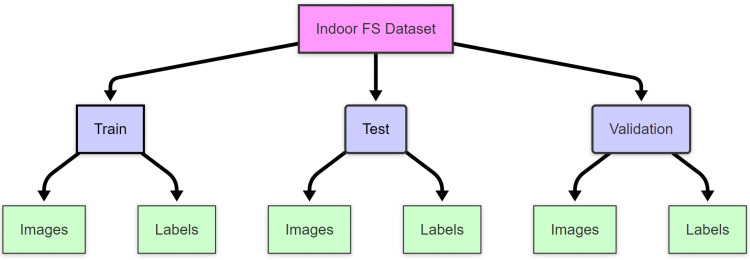
Dataset folder structure.

The Indoor FS dataset follows a hierarchical organization with three main folders: train (for model training), test (for final evaluation), and valid (for interim validation during training). Within each of these folders, there are two subfolders: one for images and another for labels, where the image files (.jpg) are stored separately from their corresponding annotation files (.txt). This structure is illustrated in Fig 1.

Auto-Orient and Resize were the preprocessing steps that were specifically applied for indoor fire and smoke detection dataset. In order to maximize the training process and improve the detection system’s performance, these transformations were implemented across all images. This dataset created a solid foundation and resource for developing and evaluating indoor fire and smoke detection systems in the future.

### 3.2 The proposed optimized YOLOv5

**Fig 2:**
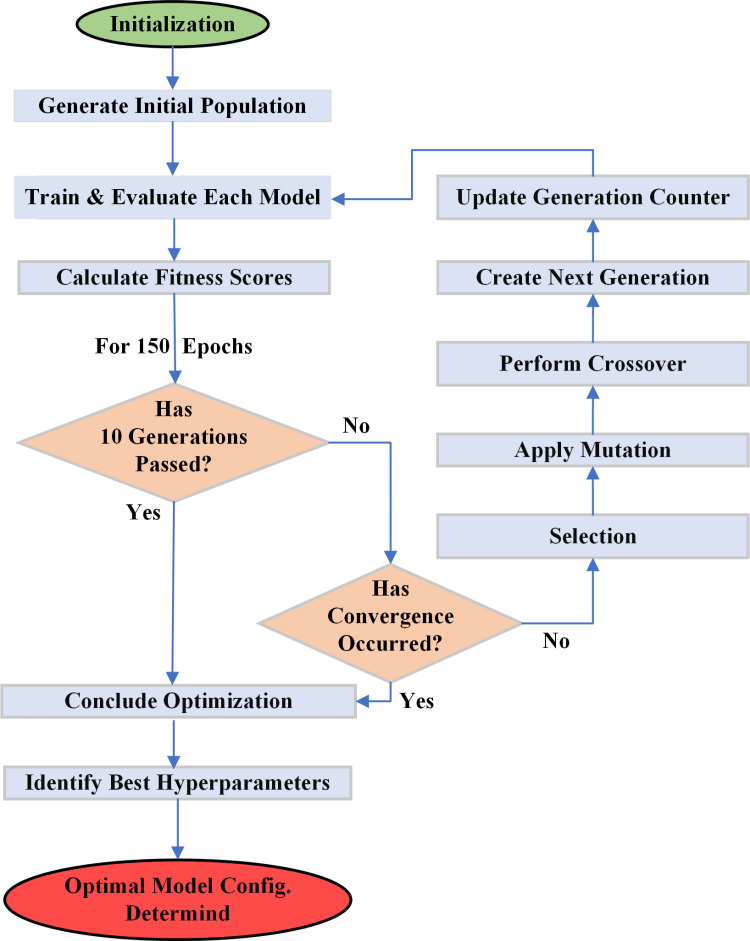
Genetic algorithm for hyperparameter optimization.

The traditional YOLOv5 architecture and its description have been documented in the literature [43]. This section describes the hyperparameter optimization of the YOLOv5 network structure via GA. The process of optimizing the baseline model is shown in the flowchart (Fig 2). The process starts by generating an initial population of network configurations. Each configuration has a unique set of thirty-two hyperparameters, randomly sampled within predefined ranges based on a seed network setup. These hyperparameters include learning rate, momentum, weight decay, batch size, augmentation settings, etc. After generating the initial population, each model was trained for 150 epochs to ensure stable performance evaluation [66]. Post-training, the models were evaluated using fitness scores where mAP@0.5 is the primary evaluation metric. Then, the GA refined the models through selection, crossover, and mutation across multiple generations. The initial population consisted of 20 candidate models (N=20) and was selected accordingly. This number was chosen based on empirical studies to balance efficiency and performance. The top 50% of models from each generation were ranked by fitness scores. They were selected for the next generation. This ensured that well-performing models were retained, while underperforming models were discarded. “Crossover” was applied with a probability of 0.8 (P_c_ = 0.8), using a one-point crossover method to combine hyperparameters from two parent models. It created new offspring while maintaining key characteristics of high-performing models. Then, “Mutation” operation was applied with a probability of 0.2 (P_m_ = 0.2) introducing slight random changes in selected hyperparameters. This helped in preventing premature convergence and enhanced model diversity. The optimization process went through for a maximum of 10 generations or until convergence, defined as no significant improvement (≤0.5% mAP@0.5 gain) for three consecutive generations. To validate the GA settings, multiple experiments were conducted with varying population sizes (N = 10, 20, 30), crossover probabilities (0.5, 0.8, 1.0), and mutation probabilities (P_c_ = 0.1, 0.2, 0.3). The chosen values (N = 20, P_c_ = 0.8, P_m_ = 0.2) provided the best tradeoff between convergence speed and accuracy. Increasing the population size beyond 20 resulted in diminishing returns in accuracy while significantly increasing computation time. Similarly, mutation probabilities higher than 0.2 introduced more randomness, leading to unstable training. This systematic hyperparameter tuning approach allowed the genetic algorithm to effectively explore the hyperparameter space. The result is significant improvements to the detection performance of YOLOv5 model specifically for indoor fire and smoke scenarios.

### 3.3 Evaluation metrics

To objectively assess the performance of the models, this research employs precision, recall, mAP@0.5, and the F1 score. These performance metrics are significant for providing a thorough evaluation of the model’s outcomes in various aspects of prediction accuracy and reliability. We adopted the evaluation metrics used in PASCAL VOC [67] to assess our results. The formulas are written as follows (1–5).


$$Precision\;=\;\frac{TP}{TP\;+\;FP\;}$$ (1)

$$Recall\;=\;\frac{TP}{TP\;+\;FN\;}$$ (2)

$$AP\;=\;\int_{0}^{1}P\;(rdr$$ (3)

$$mAP\;=\;\frac{\sum_{i=1}^{k}AP_{i}}{k}$$ (4)

$$F1\;=\;2*\frac{(Precision\;*\;Recall}{(Precision\;+\;Recall)\;}$$ (5)

Precision (P) measures how accurately a model predicts positive instances, calculated as the ratio of true positives (TP) to the total predicted positives (TP + FP), expressed as in Eqn. (1). Recall (R) assesses the model’s ability to detect all relevant positive instances, calculated as the ratio of true positives to the total actual positives (TP + FN) (Eqn. (2)). Average Precision (AP) is the area under the Precision-Recall curve for a specific class, summarizing the trade-off between precision and Recall. Mean Average Precision (mAP) extends AP to multiple classes by averaging AP values across classes (Eqn. (3)). Specifically, mAP@0.5 refers to the mean average precision calculated with an Intersection over Union (IoU) threshold of 0.5, meaning a predicted bounding box is considered a true positive if it overlaps with the ground truth by at least 50%. A higher IoU threshold (e.g., 0.75) requires more precise predictions to be considered correct, while a lower threshold (e.g., 0.5) allows for more flexibility. A more lenient threshold (e.g., 0.5) was sufficient for broader detection tasks, where detecting the presence of a fire or smoke was more important than pinpointing the exact location.

The F1-score balances precision and Recall, particularly useful for imbalanced classes (Eqn. (5)). In these formulas, TP (true positives) are correctly predicted positive samples, TN (true negatives) is correctly predicted negative samples, FP (false positives) are negatives misclassified as positives, and FN (false negatives) are positives misclassified as negatives. The Precision-Recall curve (P(r)) showed the trade-off between precision and recall at different thresholds, and k represents the number of classes. By utilizing these metrics, this study ensures a thorough and balanced evaluation of the performance of the model.

### 3.4 Explainable AI Methods for Model Transparency: Grad-CAM

**Fig 3:**
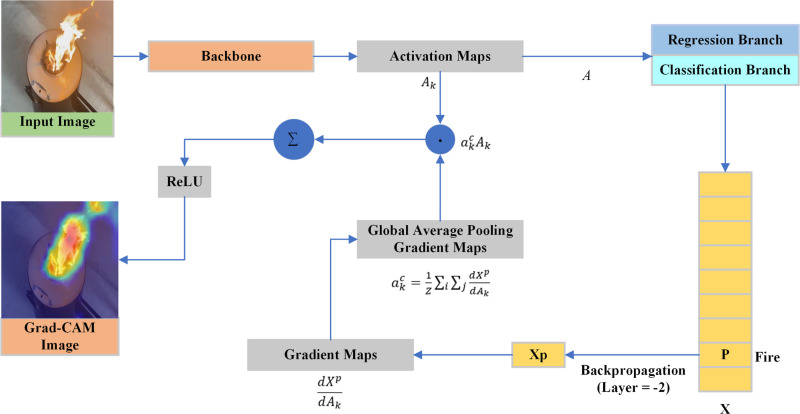
XAI (Grad-Cam) architecture.


Explainable artificial intelligence (XAI) seeks to make the internal structure of DL models transparent and accessible to humans. Grad-CAM is an increasingly used XAI technique for visualizing and interpreting DL models, particularly CNNs. This study used the Grad-CAM method to guarantee the transparency and interpretability of the HPO-YOLOv5 model. Visualizing and understanding the decisions taken by CNNs becomes much easier using this approach. Using the gradient information that reached the last convolutional layer of the CNN, this approach gives each neuron importance ratings for a specific decision of interest. This clarified which areas of an image were crucial for predicting an object via a model. Fig 3. shows the Grad-CAM technique in detail by block. The penultimate convolutional layer (layer = -2) of the model explains the decisions made at the output layer. It starts with an input image that the model is supposed to classify or analyze. For instance, the image might depict a scene of fire, which the model needs to detect. The input image is then passed through the backbone of a CNN, which consists of multiple layers of convolutional filters. These layers transform the input image into high-level feature maps, capturing various aspects and patterns present in the image. The convolutional layers produce activation maps ($A_{k}$), which are intermediate outputs that represent different features detected in the input image. Each activation map highlights specific features such as edges, textures, or shapes. Grad-CAM requires the computation of gradients of the target class score with respect to the activation maps. This is done through backpropagation from a particular layer (often the last convolutional layer) to compute the gradients, ($\frac{dy^{c}}{dA_{k}}$) where $Y^{c}$ is the score for class c. The gradients are globally averaged over the spatial dimensions (width and height) to obtain the important weights ($a_{k}^{c}$) these weights indicate the importance of each feature map in the final decision for the target class [58].

$$a_{k}^{c}=\;\frac{1}{Z}\;\sum_{i}\sum_{j}\frac{\partial Y^{c}}{\partial A_{k}^{ij}}$$ (6)

The importance weight ($a_{k}^{c}$), is given by Eqn. (6), are used to weigh the corresponding activation maps, where Z is the number of pixels in the activation map and are the spatial locations. These weighted activation maps are then summed to produce a coarse localization map, $L_{Grad-CAM}^{c}$, that highlights the regions in the image relevant to the target class [58].


$$L_{Grad-CAM}^{c}=ReLU\;(\sum_{k}a_{k}^{c}\;A_{k}\backslash )$$ (7)

A ReLU (Rectified Linear Unit) activation function is applied to the coarse localization map to retain positive contributions, focusing on regions that influence the target class prediction. The resulting Grad-CAM heatmap highlights key parts of the input image that are important to the model’s decision, providing a visual explanation. This is crucial for identifying which image sections the model focuses on when predicting smoke and fire. In the case of fire detection, the Grad-CAM heatmap should indicate the fire regions, indicating that the model’s focus is consistent with human intuition. Similarly, for smoke detection, the heatmap should include areas with visible smoke. The adoption of Grad-CAM in our proposed model improves interpretability by providing insights into the decision-making process of the model. This transparency is critical for applying the model in real-world safety and monitoring systems because understanding the foundation of its predictions can increase trust and allow for more effective responses.

### 3.5 Concept of Fire hazard detection

Detecting fire hazards accurately is crucial because traditional detection methods can sometimes produce false positives, causing false alarms. For instance, objects with similar visual characteristics to fire, such as a yellow t-shirt, might be incorrectly identified as a fire. Similarly, small, controlled fires, like those from a candle, may not represent a significant danger. Therefore, to reliably detect actual fire hazards, it is necessary to monitor the progression of the fire over time. By monitoring how a fire grew or changed over time, the system dynamically assessed and differentiated between non-threatening situations and actual fire hazards. Continuous tracking allows for real-time updates, ensuring that safety measures are activated, when necessary, thus reducing unnecessary alarms. This section outlines an overall approach that utilizes state-of-the-art object detection and tracking algorithms to infer potential fire hazards from video frames. The methodology involves the use of YOLOv5 for object detection and DeepSORT for object tracking, followed by comparisons to identify significant changes in fire size.

**Fig 4:**
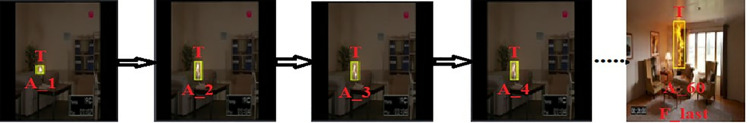
Fire monitoring process.

In Fig 4, a real-world video with a duration of approximately 4 minutes was used, and a 60-second segment was selected for analysis. This segment was sampled at a rate of 1 frame per second (fps), yielding a total of 60 frames. The original video, recorded at 30 frames per second, was downsampled to 1 fps using OpenCV [66] for further processing. The methodology began by detecting and inferring potential fire hazards in the video captured at 1 fps over the 60-second interval. First, the YOLOv5 model detected objects in each video frame and outputted bounding boxes (B) around the fire instance. We only considered here a single fire instance (T_1_). The DeepSORT algorithm tracked T_1_ across the frames, keeping a consistent identification of the fire instance. In the first frame, the initial bounding box area (A_1_) was calculated, which served as the baseline for further comparisons. A running sum (S_A_) is then initialized to track the total area across frames. From frame 2–60, the bounding box area (A_t_) is calculated for each frame. This value was added to the running sum (SA). All 60 frames were processed, the average bounding box area (A̅) was computed using the formula, A̅ = S_A_/ t - 1, to efficiently calculate the average. In the last frame (Frame no. 60), the total bounding box area (B_total_) was determined. The average area for the last frame (B̅) was also calculated. The algorithm then compared the initial area (A1) to A̅ and B̅. If either A̅ - A1 or B̅ - A_1_ exceeds a set threshold (U), a fire hazard incident was detected (H = True). If not, no significant fire growth was observed (H = False). Finally, the algorithm outputs the status of the fire hazard inference (H).

**Pseudo Algorithm for Fire Hazard Inference (1 fps, Single Instance)**

A. Object Detection (YOLOv5):

- **Input:** Video frames, F (1 fps)
- **Output**: Detected bounding boxes, B
- **Process: B** = YOLOv5(F)

B. Object Tracking (DeepSORT):

- **Input**: Detected bounding boxes, B
- **Output**: Tracked fire instance, T (T1)
- **Process**: T = DeepSORT(B)

C. Initial Area Calculation (Frame 1):

- **Input**: Tracked fire instance, T_1_
- **Output**: Initial bounding box area, A_1_
- **Process**:
- A_1_ = Area (B_initial_ (T_1_))
- Initialize S_A_ = 0 (Running sum for instance T_1_)

D. Monitoring (Frame 2 to 60):

- **Input**: Tracked fire instance T, Time interval Δt = 60 s
- **Output**: Average area A̅ for T_1_
- **Process**:
- For each frame t = 2 to 60 (1 fps):
- Calculate area for T_1_ at time t:
- A_t_ = Area (T_1, t_)
- Update running sum:
- S_A_ = S_A_ + A_t_
- After 60 frames, compute the average area:
- A̅ = S_A_/ (t - 1) (Efficient mean calculation)

E. Last Frame Area (Frame 60):

- **Input**: Last frame F_last_, Tracked instance T_1_
- **Output**: Total area B_total_ and average area B̅
- **Process**:
- B_total_ = Area (F_last_ (T_1_))
- B̅ = B_total_

F. Comparison:

- **Input**: A1, A̅, B̅, U
- **Output**: H
- **Process**:

G. Final Output:

- Fire hazard inference H.

This approach combines object detection, tracking, and dynamic area calculations to develop a strong framework for real-time fire hazard detection.

## 4 Experimental results and discussion

### 4.1 Dataset description

The Indoor Fire and Smoke Dataset is a carefully labelled collection of images with a median resolution of 416 by 416 pixels. The dataset distribution comprises a total of 5,000 images and 5,931 instances, with a split of 80% for training, 10% for validation, and 10% for testing, as elaborated in Table 1. Since an image may have more than one instance, the total image samples (*) have not been calculated.

**Table 1:** Dataset distribution by class.

Class	Number of Images					Number of Instances		
	Training	Validation	Testing	Total	Training	Validation	Testing	Total
Fire	2009	290	221	2520	2571	299	251	3121
Smoke	2195	236	279	2710	2290	241	279	2810
Total	4204	526	500	*	4861	540	530	5931

**Fig 5:**
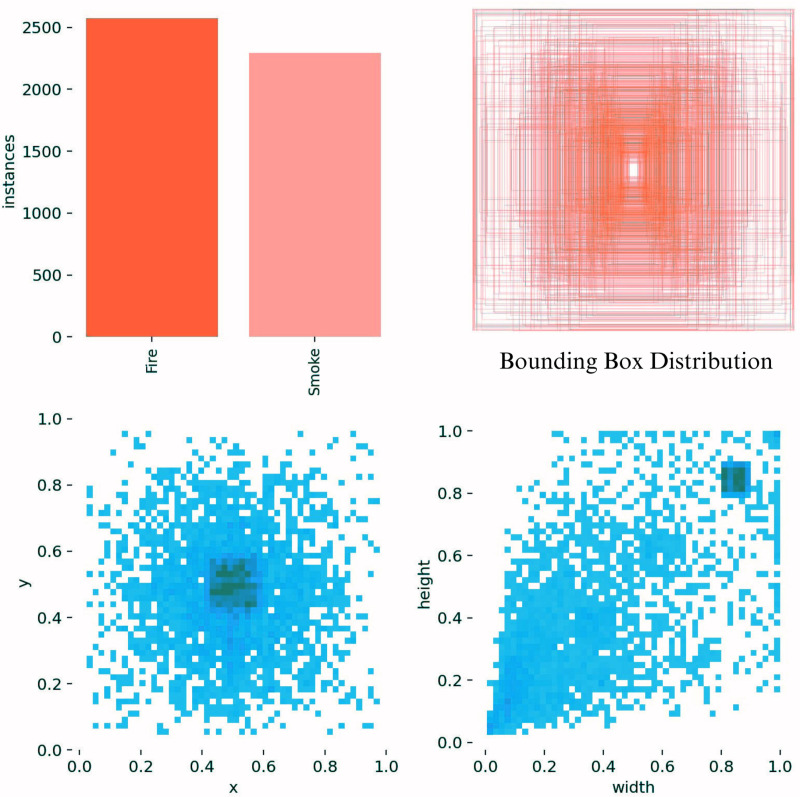
Statistical analysis of indoor fire and smoke dataset labels.

To analyze whether the dataset utilized in this research corresponded to the distribution of fire and smoke instances in indoor environments, a statistical analysis of the dataset labels was implemented, and the outcomes were presented in Fig 5. The bar chart suggests that the number of ‘Fire’ instances is greater than that of ‘Smoke’. The central region of the image exhibits a higher density in the distribution of bounding box sizes. The scatter plots revealed that most labels were centered, with a concentration of the ratio of horizontal and vertical coordinates in the center of the labels. Furthermore, the label width to image width and label height to image height ratios indicate that the majority of label frames are of comparable dimensions. This broad distribution, which was primarily concentrated in the center of the image, suggested that the dataset contained small to large-sized objects, which were consistent with actual indoor fire and smoke scenarios.

### 4.2 Experiment platform

The training of the proposed model was conducted using an image size of 416 by 416 pixels, a batch size of 8, and over 100 epochs. For training, the experimental environment settings are described in Table 2.

**Table 2:** The experimental environment settings.

Items	Type
Operating System	Ubuntu 18.04.3
Deployment environment	Python 3.10.12
Memory	16GB of HBM2
CPU	Intel(R) Xeon(R) CPU @ 2.20GHz
GPU	Tesla V100 GPU
DL framework	PyTorch 2.1.0
Accelerated computing architecture	CUDA 12.1

### 4.3 Hyperparameter results

In this study, the baseline YOLOv5 model was subjected to significant hyperparameter tuning using a genetic algorithm approach in order to optimize its performance for indoor fire and detection tasks. Table 3 summarizes the key hyperparameters adjusted during our experiments, along with their key identifiers, descriptions, and magnitudes.

**Table 3:** Hyperparameters characteristics.

Parameter	Identifier	Description	Magnitude
*Model Hyperparameters*			
Initial Learning Rate	lr0	Starting learning rate	0.01
Final Learning Rate	lrf	Ending learning rate	0.01
Momentum	momentum	Gradient acceleration	0.98
Weight Decay	weight_decay	Regularization penalty	0.00051
*Training Hyperparameters*			
Warmup Epochs	warmup_epochs	Initial LR ramp-up period	2.6604
Warmup Momentum	warmup_momentum	Initial momentum adjustment	0.8
Warmup Bias Learning Rate	warmup_bias_lr	Bias LR during warmup	0.09617
Box Loss Weight	box	Box regression weight	0.04702
Class Loss Weight	cls	Classification loss weight	0.39525
Class Prediction Weight	cls_pw	Classification loss modifier	1.0
Objectness Loss Weight	obj	Objectness loss weight	1.187
Objectness Prediction Weight	obj_pw	Objectness loss modifier	1.0968
IOU Threshold	iou_t	IoU detection threshold	0.2
Anchor Threshold	anchor_t	Anchor matching threshold	4.0
*Augmentation Hyperparameters*			
Hue Adjustment	hsv_h	Hue shift	0.01717
Saturation Adjustment	hsv_s	Saturation shift	0.55958
Value Adjustment	hsv_v	Brightness shift	0.43654
Translation	translate	Image translation	0.0974
Scaling	scale	Image scaling	0.45796
Flip Left-Right Probability	fliplr	Horizontal flip chance	0.5
Mosaic	mosaic	Mosaic augmentation	0.973

### 4.4 Analysis of HPO-YOLOv5 experimental results

Post-training, the model comprised 157 layers, 7,015,519 parameters, and zero gradients, completing the training process in 2.401 hours. The outcomes showed significant performance improvement, as seen in Table 4.

**Table 4:** Proposed model performance by class.

Class	P (%)	R (%)	F1 (%)	mAP@0.5 (%)
All	92.7	85.5	88.78	92.1
Fire	88.0	88.1	87.95	90.6
Smoke	97.4	83.0	89.47	93.6

**Fig 6:**
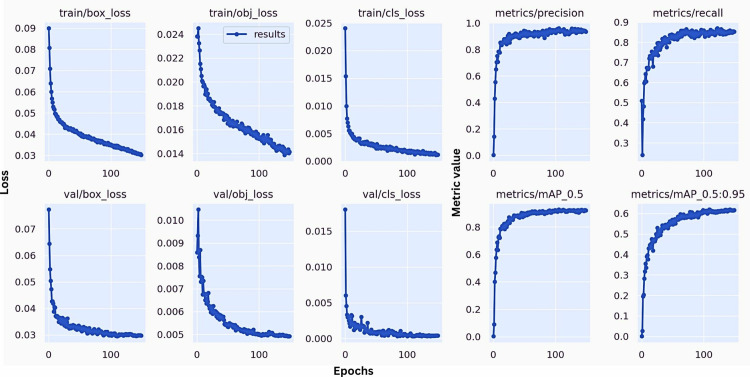
Improved YOLOv5s training results.

**Fig 7:**
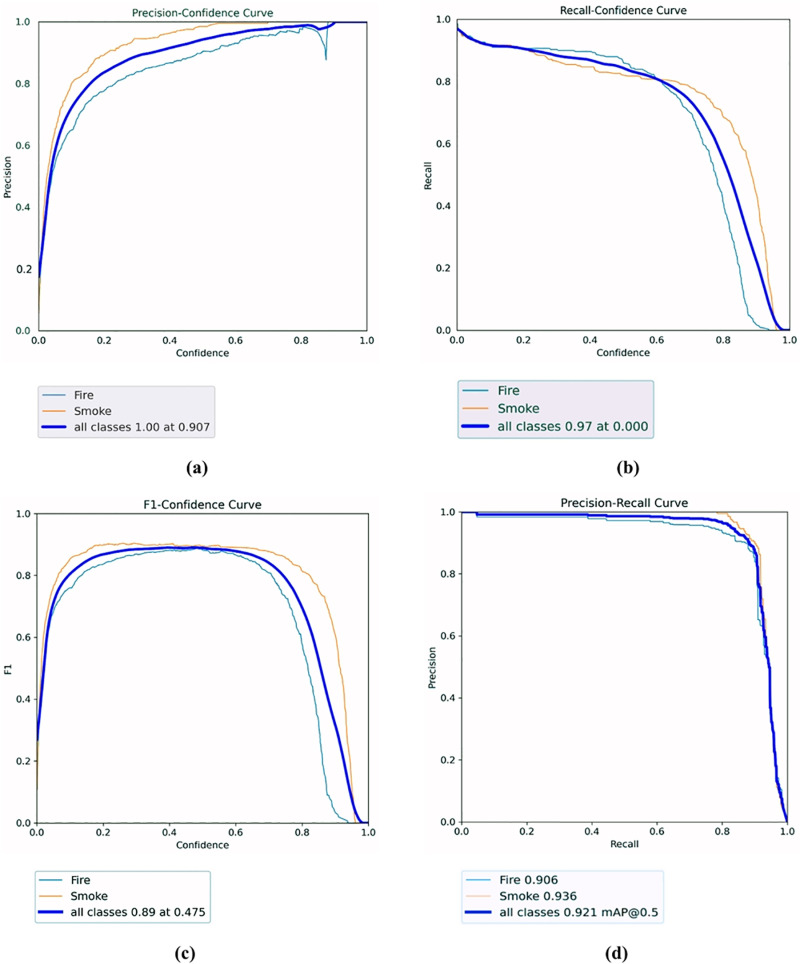
Training progression curves: (a) Precision; (b) Recall; (c) F1; (d) P-R.

The training process of the HPO-YOLOv5 model is depicted through a series of loss and performance metric curves, which highlight the learning dynamics and efficacy of the model. As shown in Fig 6, the training and validation losses for box regression, objectness, and classification steadily decrease, indicating effective learning and convergence over the epochs. Post-training evaluation metrics display significant improvements. The Precision-Confidence Curve (Fig 7(a)) shows high precision across various confidence levels, with the smoke class achieving higher precision than the fire class. The Recall-Confidence Curve (Fig 7(b)) illustrates that recall remains high across different confidence thresholds, though the fire class has slightly lower recall compared to the smoke class. The F1-Confidence Curve (Fig 7(c)) balances precision and recall, showing the optimal confidence threshold for peak performance. Finally, the Precision-Recall (P-R) Curve (Fig 7(d)) displays the ability of the model to maintain high precision and recall across various thresholds, with the smoke class generally outperforming the fire class. These curves collectively validate the robustness and accuracy of our HPO-YOLOv5 model in detecting fire and smoke, highlighting its potential for reliable indoor safety and monitoring applications.

**Fig 8:**
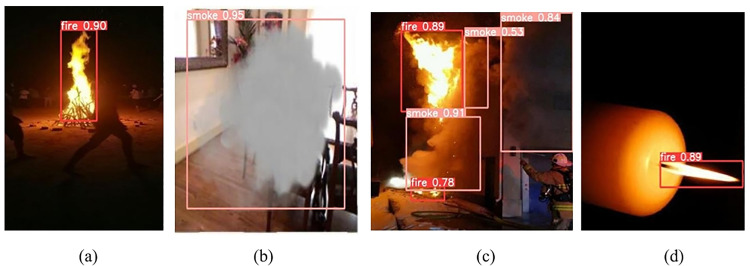
Performance in varied settings: (a) Night environment; (b) Confined spaces; (c) Complex scenario; (d) Dark environment.

The improved model HPO-YOLOv5 was evaluated on several unseen test images to assess its detection capabilities for fire and smoke in various scenarios (Fig 8). The model successfully detected a bonfire in a dark environment with a high confidence score of 90% (Fig 8(a)). It effectively identified smoke in an indoor setting with a confidence score of 95% (Fig 8(b)), presenting its ability to detect smoke accurately within confined spaces. In a complex scenario involving a fire outbreak, the model demonstrated multiple detections with varying confidence scores for both fire (89%, 78%) and smoke (91%, 84%, 53%) ((Fig 8(c)), highlighting its robustness in multi-object detection scenarios. Additionally, the model identified a small candle fire with a confidence score of 89% ((Fig 8(d)), indicating its sensitivity to detecting small-scale fires. These results indicate the proposed model’s capability to identify fire and smoke across different surroundings and conditions.

### 4.5 Analysis of experimental results of different models

The state-of-the-art (SOTA) comparison experiment was executed to assess and compare the performance of various leading object detection models, including Faster R-CNN, YOLOv5s, YOLOv7, YOLOv8n, and our HPO-YOLOv5. The performance measures analyzed were precision (P), recall (R), F1 score, mean average precision (mAP@0.5) at an IoU threshold of 0.50, and inference time (in milliseconds). The results are summarized in Table 5. To maintain fairness, all networks underwent an identical fine-tuning process during the experiment. We used an image resolution of 416 by 416, trained for 100 epochs with a batch size of 8, employed the SGD optimizer, set a patience value of 100, and a learning rate of 0.01. To reduce the impact of software and hardware on model inference time, the experiments were conducted in a controlled setup detailed in Table 2.

**Table 5:** Comparative Analysis of SOTA object detection model performance metrics.

Methods	P (%)	R (%)	F1 (%)	mAP@0.5 (%)	Inference time (ms)
Faster R-CNN	91.4	78.0	84.1	86.2	9.9
YOLOv5s	92.8	82.9	87.3	89.7	6.5
YOLOv7	89.3	81.6	85.3	88.0	7.3
YOLOv8n	91.9	83.1	87.5	90.1	6.3
Our HPO-YOLOv5	92.7	85.5	88.9	92.1	6.2

Faster R-CNN offered high precision at 91.4%, but it fell short in recall (78.0%) and had a slower inference time of 9.9 ms per instance. This highlighted a trade-off between accuracy and speed. On the other hand, YOLOv5s improved the detection with a recall of 82.9%, an F1 score of 87.3%, and an inference time of 6.5 ms, making it more efficient for real-time scenarios. YOLOv7 provided balanced performance with a precision of 89.3%, an F1 score of 85.3%, and a moderate inference time of 7.3 ms, though it did not quite match its successors. YOLOv8n performed strongly with precision and recall figures of 91.9% and 83.1%, respectively, an F1 score of 87.5%, and a quicker inference time of 6.3 ms, showcasing its robust efficiency. However, the standout performer was the HPO-YOLOv5 model. Through hyperparameter optimization, it achieved the highest recall (85.5%), the best F1 score (88.9%), and the top mAP@0.5 at 92.1%, with an inference time of just 6.2 ms.

The comparative analysis underscores the proficiency of the HPO-YOLOv5 model in object detection tasks, particularly for indoor fire and smoke detection. Its good performance in precision, recall, F1 score, and mAP, combined with low inference time, positions it as a highly effective solution for real-time safety and monitoring applications.

### 4.6 Model explainability

To explain the effectiveness of the Grad-CAM method, we present several outputs that highlight the regions in the input images on which the model focused for its predictions.

**Fig 9:**
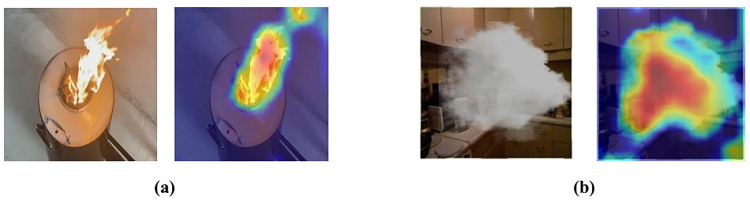
Grad-CAM heatmap detection: (a) Fire; (b)Smoke.

In the first pair of Fig 9(a), we observe the original input image of a fire and its related Grad-CAM heatmap. The input image shows a visible fire, and the Grad-CAM heatmap is superimposed on the original image, indicating the locations that the model used to make its prediction. The heatmap clearly emphasizes the fire zone, indicating that the model’s attention is correctly focused on the relevant features in the image. The highlighted regions in the Grad-CAM heatmap correspond precisely to the fire, providing the model’s ability to detect actual fire objects. In the second pair of Fig 9(b), we analyze the original input image of smoke and its related Grad-CAM heatmap. The input image displays visible smoke in a kitchen environment, and the Grad-CAM heatmap highlights the areas with visible smoke. The heatmap shows the model’s focus on the smoke regions, indicating its ability to correctly identify and prioritize these features for prediction. The Grad-CAM heatmap highlights the regions containing smoke objects, validating that the model effectively recognizes and focuses on the smoke regions within the image.

The aforementioned cases show that the HPO-YOLOv5 effectively focuses on relevant features, like fire in fire detection and smoke in smoke detection.

### 4.7 Fire hazard experimental outputs

The experimental methodology for fire hazard detection starts with the initialization phase, where object detection is carried out using YOLOv5 to identify fire instances in video frames, and object tracking is managed using DeepSORT to provide consistent IDs for these instances across frames. For further frame-based analysis, OpenCV (Open-Source Computer Vision Library) [68] is used here.

**Fig 10:**
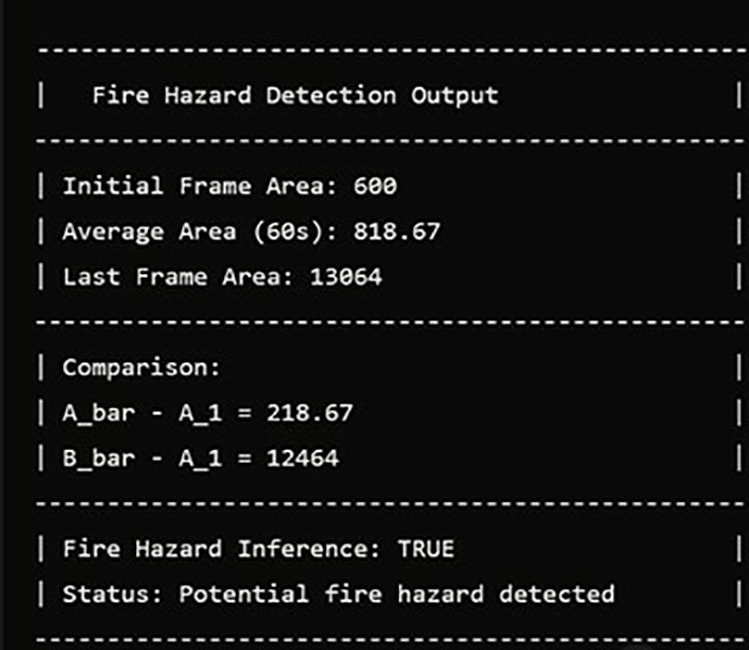
Fire hazard detection output.

The final output, shown in Fig 10, follows the fire hazard detection methodology across video frames. It presents the initial bounding box area from frame 1, the average area over frames 2–60, the last frame’s area, and the fire hazard inference. In here, the initial area is 600 square pixels, the average area is 818.67 pixels, and the last frame area is 13,064 pixels. These values are compared to a threshold (U = 1000) to assess fire growth. The system calculates the average bounding box area over 59 frames and compares it to the initial area. Although the average difference (218.67) does not exceed the threshold, the difference in the last frame (12,464) far exceeds the threshold, indicating Fire Hazard Inference: True and potential fire hazard detected status.

**Threshold (U)** We have historical data via experiments showing that typical non-hazardous fire instances have bounding box areas between 500 and 1500 square pixels. Bounding box areas have been observed to increase by 2000–5000 square pixels during hazardous conditions. Primary, a threshold of 1000 is set, as it is above normal fluctuations (0–400) and aligns with significant fire growth patterns. This threshold balances sensitivity (detecting real hazards) and specificity (avoiding false alarms). If the system generates false alarms, the threshold can be increased, and if it misses hazards, it can be lowered later. This ensures the detection system is practical and reliable. The threshold can be adjusted to fine-tune sensitivity and prevent errors.

## 5 Conclusion and future works

This study focuses on indoor fire and smoke detection by developing an HPO-YOLOv5 model using a genetic algorithm and introducing a novel indoor fire and smoke dataset. It addresses the limitations of traditional fire detection methods by image-based techniques, resulting in high mAP@0.5 (92.1%) and recall (85.5%). The integration of the Grad-CAM technique ensures model transparency and interpretability, while the combination of YOLOv5 with DeepSORT assists real-time monitoring of fire progression, providing early fire hazard warnings. Comparative analysis with SOTA models, including Faster R-CNN with Detectron2, YOLOv5s, and YOLOv7, shows that the HPO-YOLOv5 model has more reliable performance than others in terms of accuracy and robustness. Future work may focus on expanding the indoor fire-smoke dataset, exploring advanced model architectures, implementing real-world deployments, enhancing explainability, and predicting fire hazard detection in more real use cases. DeepSORT may struggle with dynamic fire changes, such as rapid alterations in fire shape and size, leading to tracking failures. These challenges can be mitigated by using more sophisticated tracking algorithms or supplementary sensors in the future. These efforts aim to improve the detection system performance, enhance practical use, and increase safety in indoor environments.
